# Mitigating bias in multilabel medical text classification: a cooperative training framework with dynamic debiasing

**DOI:** 10.1093/bioinformatics/btag317

**Published:** 2026-05-18

**Authors:** Pengfei Li, Xu Zhang, Xiaoyu Hu, Zexu Lin, Deyu Zhou

**Affiliations:** School of Computer Science and Engineering, MOE Key Laboratory of Computer Network and Information Integration, Southeast University, Nanjing, Jiangsu 210096, China; School of Computer Science and Engineering, MOE Key Laboratory of Computer Network and Information Integration, Southeast University, Nanjing, Jiangsu 210096, China; School of Computer Science and Engineering, MOE Key Laboratory of Computer Network and Information Integration, Southeast University, Nanjing, Jiangsu 210096, China; School of Computer Science and Engineering, MOE Key Laboratory of Computer Network and Information Integration, Southeast University, Nanjing, Jiangsu 210096, China; School of Computer Science and Engineering, MOE Key Laboratory of Computer Network and Information Integration, Southeast University, Nanjing, Jiangsu 210096, China

## Abstract

**Motivation:**

Medical text classification plays a critical role in clinical decision support, automated diagnosis, and biomedical research. However, deep learning models are highly susceptible to dataset-induced biases, such as label bias and keyword bias, which can lead to unreliable predictions and poor generalization in real-world clinical applications. Existing debiasing methods often either overcorrect informative samples or lack interpretability during inference, limiting their effectiveness in multilabel medical text classification tasks.

**Results:**

We propose Cooperative Debiasing Network (CoDeNet), a cooperative training framework that mitigates dataset bias through dynamic sample reweighting and interpretable counterfactual inference. The framework consists of a primary classifier and a debias estimator, where the debias estimator quantifies sample-level bias and dynamically regulates the optimization process through an elastic scaling mechanism. In addition, a counterfactual postprocessing strategy explicitly isolates label-level and keyword-level biases to improve interpretability. Experiments conducted on the DepressionEMO and BDI-Sen datasets demonstrate that CoDeNet consistently improves classification performance over strong transformer-based baselines, including BERT and MentalBERT. In particular, CoDeNet achieves improvements of up to **+6.57% macro-F1 on BDI-Sen** and **+2.16% macro-F1 on DepressionEMO**, with especially strong gains on low-frequency clinical labels. The results indicate that CoDeNet effectively reduces dataset-induced bias while preserving model robustness and interpretability.

**Availability and implementation:**

The source code and implementation details of CoDeNet will be publicly available on GitHub: https://github.com/66ccff39C5BB/CoDeNet.

## 1 Introduction

Medical text classification represents a fundamental task in biomedical informatics, serving as the foundation for applications such as clinical decision support, automated diagnosis, and large-scale biomedical literature analysis (Rajkomar *et al.* 2018, Ceccarelli *et al.* 2023, Chen *et al.* 2025). Recent advancements in deep learning have yielded state-of-the-art performance across various medical text classification benchmarks, demonstrating their efficacy in handling complex biomedical data (Prabhakar and Won 2021, Lu *et al.* 2022, Li *et al.* 2024). However, despite their success, deep learning models remain vulnerable to biases inherent in training data, leading to unreliable predictions and diminished generalizability (Varghese *et al.* 2019, Schmidgall *et al.* 2024).

Bias in medical text classification often stems from spurious correlations present in the dataset, including label bias and keyword bias (Zhang *et al.* 2020). Label bias arises when certain labels are overrepresented or misrepresented due to systemic inconsistencies in data collection, while keyword bias occurs when models disproportionately rely on specific terms rather than contextual semantics (Jiang and Nachum 2020, Waldeyer and Roelle 2021). Such biases can result in erroneous model predictions, posing significant risks in high-stakes clinical applications where misclassification may lead to severe consequences (Char *et al.* 2018, Challen *et al.* 2019). Therefore, addressing these biases is imperative to enhancing the robustness and fairness of deep learning models in biomedical applications.

To mitigate these challenges, various debiasing techniques have been explored, including counterfactual data augmentation (Pitis *et al.* 2022, Temraz and Keane, 2022), adversarial training (Yang *et al.* 2023), and sample reweighting strategies (Borland *et al.* 2021). Counterfactual data augmentation seeks to artificially balance training data distributions; however, it often introduces noise and increases computational complexity (Cao *et al.* 2024). Adversarial training approaches aim to learn invariant feature representations to reduce bias, yet they risk suppressing clinically meaningful signals (Min and Vidal 2024). Sample reweighting techniques adjust the influence of biased instances, though existing approaches struggle to distinguish genuine correlations from spurious ones (Keller and Bolhuis 2024). Notably, Junhyun *et al.* introduced a debiasing approach known as Learning from Failure, which trains a pair of neural networks simultaneously to address dataset bias, offering novel insights into model debiasing (Nam *et al.* 2020). Additionally, Chen *et al.* proposed CORSAIR, a counterfactual reasoning-based framework for debiasing text classification models, effectively mitigating dataset-induced biases (Qian *et al.* 2021).

However, these approaches remain limited when applied to multilabel medical text classification. Dynamic sample reweighting methods can adaptively adjust learning signals and have shown potential for bias mitigation, yet they typically focus only on aggregate training trends and lack explicit reasoning about how bias manifests in individual examples at inference time. Conversely, counterfactual methods simulate hypothetical bias at inference time, but they are often applied as isolated postprocessing steps without incorporating data-driven debiasing signals learned during training.

Motivated by the complementary strengths of these two paradigms, we propose the Cooperative Debiasing Network (CoDeNet), a cooperative training framework aimed at mitigating biases in deep learning-based medical text classification. CoDeNet comprises two interdependent components: a primary classifier, responsible for learning unbiased and clinically meaningful representations, and a debias estimator, which identifies and mitigates spurious correlations by quantifying biases.

A key innovation of our approach is the integration of an elastic scaling mechanism within the debias estimator, enabling dynamic adjustment of bias weights to prevent overcorrection and preserve model efficacy. The intuition is that samples already predicted with high confidence often contain cleaner, more reliable patterns and less annotation noise—particularly in noisy clinical text corpora where label uncertainty and symptom ambiguity are common. By amplifying the learning signal from such samples, the model is encouraged to extract high-quality features that generalize beyond spurious correlations. Furthermore, we incorporate counterfactual inference to interpretably enhance model performance, facilitating the identification of biased features and improving transparency in medical artificial intelligence applications. We conduct comprehensive evaluations on benchmark multilabel medical text classification datasets, demonstrating that CoDeNet effectively reduces bias-induced distortions, leading to improved classification performance, generalization, and interpretability.

This work presents the design, implementation, and empirical evaluation of CoDeNet. The primary contributions are as follows:

1. A cooperative training framework for debiasing multilabel medical text classification.
2. An elastic scaling mechanism with a debias estimator with for dynamically regulating bias correction.
3. The integration of counterfactual inference to interpretably improve model performance.
4. Extensive experimental validation on benchmark datasets,showcasing the efficacy of CoDeNet in enhancing classification accuracy and robustness. Our source code will be publicly available on GitHub (https://github.com/66ccff39C5BB/CoDeNet).

The remainder of this paper is structured as follows. Section 2 describes the CoDeNet architecture and training methodology. Section 3 presents experimental results and analysis. Section 4 discusses the innovation, limitations and potential future directions of CoDeNet. Finally, Section 5 concludes the paper. Due to space limitations, several extended discussions are presented in the Supplementary Material, available as supplementary data at *Bioinformatics* online. These include an expanded review of related work, additional experimental results on a more widely used medical dataset, comparative evaluations against other debiasing methods, and further qualitative sample analyses.

## 2 Materials and methods

This section outlines the primary methodology proposed in this study, where a supervised model was employed to perform a multilabel text classification task. Specifically, the primary classifier, denoted as $M_{1}$ in the latter, was first trained using conventional supervised learning as a preliminary step. Following this, the debias estimator, denoted as $M_{2}$ in the latter, which shares the same structure but employs a different loss function, was introduced to assist the primary classifier in reducing biases by adapting the learning process according to the discrepancy between the two models. In addition, we incorporate a counterfactual method in the postprocessing phase, which aims to mitigate bias in the model’s decision-making process interpretably. The complete pipeline of CoDeNet is summarized in Algorithm 1, which consists of three sequential stages corresponding to different functional components of the framework.

1. **Preliminary step (lines 1–5).** The primary classifier $M_{1}$ is first optimized using standard cross-entropy (CE) loss to learn initial label representations without bias correction. This step provides a stable starting point and prevents undesirable interference from the debias estimator during early training.
2. **Cooperative training (lines 6–11).** After warm-up, the debias estimator $M_{2}$ is initialized and jointly trained with $M_{1}$.
3. **Postprocessing (lines 12–18).** After model training, we apply an interpretable counterfactual adjustment to mitigate residual label and keyword bias.

### 2.1 Problem formulation

Multilabel text classification involves assigning a set of relevant labels $Y=\{y^{\left(1\right)},y^{\left(2\right)},\ldots ,y^{\left(L\right)}\}$ from a predefined label space to a given text instance *x*. Unlike single-label classification, where each instance corresponds to one label, multilabel classification allows each instance *x* to be associated with multiple labels simultaneously.

Formally, let $D={\left\{(x_{i},y_{i})\right\}}_{i=1}^{N}$ represent the dataset, where $x_{i}$ is the *i*th text instance, and $y_{i}=[y_{i}^{\left(1\right)},y_{i}^{\left(2\right)},\ldots ,y_{i}^{\left(L\right)}]\in {\left\{0,1\right\}}^{L}$ is a binary vector indicating the presence ($y_{i}^{\left(j\right)}=1$) or absence ($y_{i}^{\left(j\right)}=0$) of each label $y^{\left(j\right)}$ for the instance $x_{i}$. The goal is to learn a mapping function $f:X\to {\left\{0,1\right\}}^{L}$, where X represents the input space, such that f(x) predicts the set of relevant labels for any given instance *x*.

### 2.2 Preliminary step

To establish a robust baseline, the primary classifier was trained as a conventional supervised multilabel classification model. This section details the encoding process, classification mechanism, and optimization strategy employed during this phase. The model receives a text instance as input and encodes it using a pretrained Transformer-based text encoder. This encoder maps the input text into a dense vector representation:


(1) h=Encoder(x),

where *x* denotes the input text and h represents its encoded feature vector. The encoded representation is subsequently processed by a multilayer perceptron (MLP) classifier, which generates a probability distribution over possible labels:


(2) p=MLP(h),

where $p\in R^{L}$ is the probability vector, with each element $p^{\left(i\right)}$ indicating the confidence level for assigning label *i* to the input instance.

The model is optimized using the standard multilabel classification loss function, CE, which independently evaluates the discrepancy between the predicted probabilities and the ground-truth label vectors:


(3) $$CE(f(x;\theta ),y)=\sum_{i=1}^{L}y^{\left(i\right)}\;\text{log}\;f^{\left(i\right)}(x;\theta ),$$

where *L* represents the total number of labels, $y^{\left(i\right)}$ is the ground-truth binary label for class *i*, and $f^{\left(i\right)}(x;\theta )$ is the predicted probability for that class.

This training phase enables the primary classifier to capture essential patterns and label dependencies within the dataset, serving as a fundamental component for the subsequent steps in our framework.

### 2.3 Cooperative training

**Figure 1:**
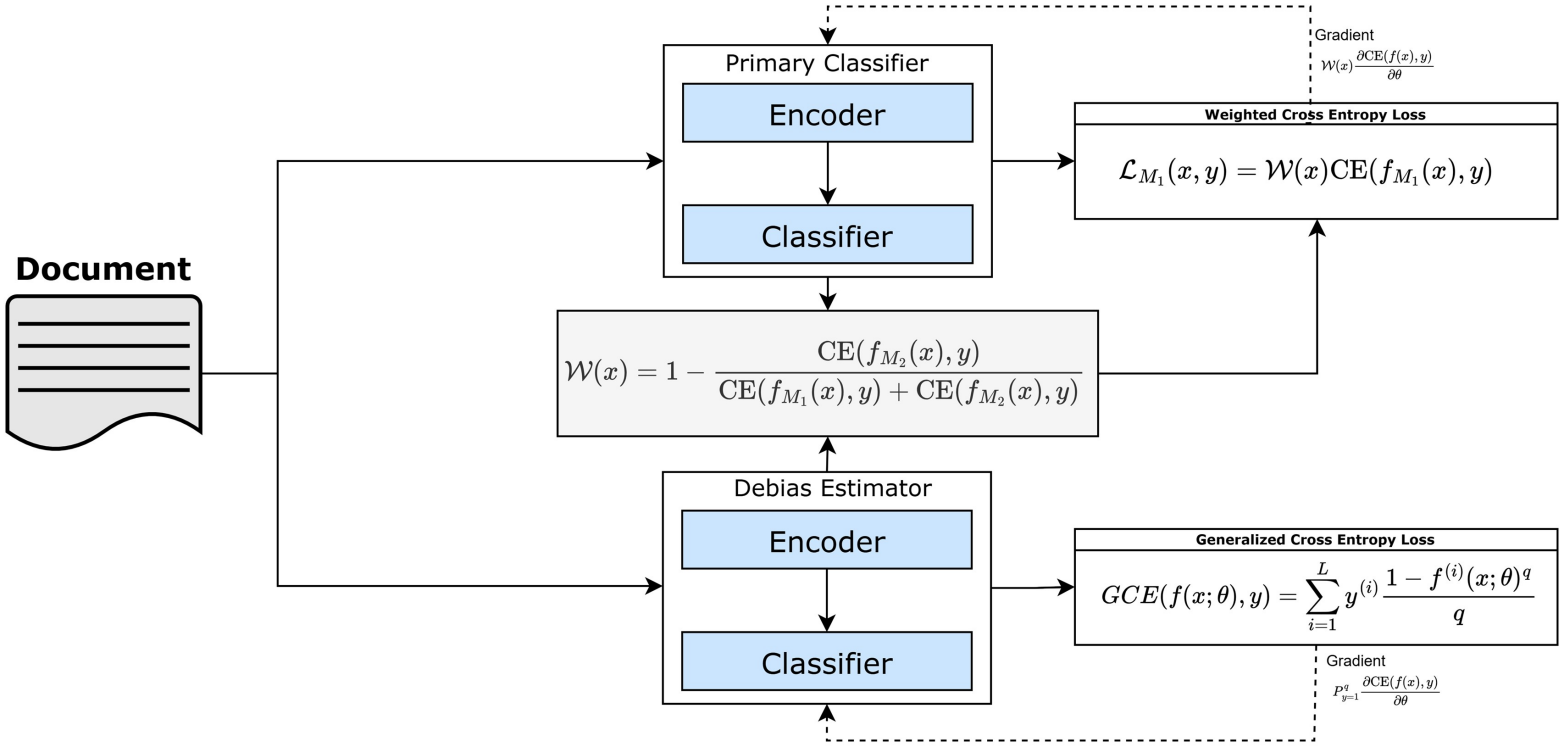
Overview of the cooperative training step, where the primary classifier (*M*_1_) and debias estimator (*M*_2_) share the same architecture but are optimized with different objectives.

Cooperative training is designed to reduce biases by dynamically adapting the learning process based on the discrepancy between two models. The approach begins with the creation of a debias estimator, which shares the same structure as the primary classifier but utilizes a different loss function, as shown in Fig. 1. In particular, the debias estimator employs the generalized cross-entropy (GCE) loss (Zhang and Sabuncu 2018), defined as follows:


(4) $$\mathrm{GCE}(f(x;\theta ),y)=\sum_{i=1}^{L}y^{\left(i\right)}\frac{1-f^{\left(i\right)}{\left(x;\theta \right)}^{q}}{q},$$

where q∈(0,1] is a hyperparameter that controls the degree of deviation of the GCE loss from the standard CE loss. The gradient of the GCE loss is a weighted version of the CE loss gradient:


(5) $$\frac{\partial \mathrm{GCE}(f(x;\theta ),y)}{\partial \theta }=\sum_{i=1}^{L}f^{\left(i\right)}{\left(x;\theta \right)}^{q}*\frac{\partial \text{CE}(f^{\left(i\right)}(x),y^{\left(i\right)})}{\partial \theta }.$$

As illustrated in Equation (5), samples for which the model assigns a higher probability to the true label receive a larger weight in the gradient update. In effect, the GCE loss prioritizes learning from more representative samples. However, GCE loss is a highly aggressive optimization method, which may lead to overcorrection and ultimately degrade overall model performance. Therefore, instead of directly employing it as the classifier, we only use it to estimate the degree of debias in the sample. When a sample is well predicted (i.e. the true label probability is high), the CE loss computed by the debias estimator is significantly lower compared to that of the primary classifier. This discrepancy is quantified by the following weight function:


(6) $$W(x)=1-\frac{\text{CE}(f_{M_{2}}(x),y)}{\text{CE}(f_{M_{1}}(x),y)+\text{CE}(f_{M_{2}}(x),y)}.$$

With this weight W(x), an elastic scaling mechanism is designed to adjust the contribution of each sample during the training of the primary classifier. Specifically, samples where the primary classifier is relatively more confident (i.e. higher predicted probabilities for true labels) receive a larger weight, whereas less confidently predicted samples contribute less. This elastic scaling mechanism is achieved by incorporating the weight W(x) into the loss function for the primary classifier as follows:


(7) $$L_{M_{1}}(x,y)=W(x)\times \text{CE}(f_{M_{1}}(x),y).$$

By leveraging the debias estimator in this auxiliary role, we mitigate the risk of excessive corrections while still benefiting from its ability to identify and emphasize more informative samples. This cooperative setup allows the primary classifier to refine its predictions in a more balanced manner, reducing bias without compromising overall classification performance.

### 2.4 Post-processing

**Figure 2:**
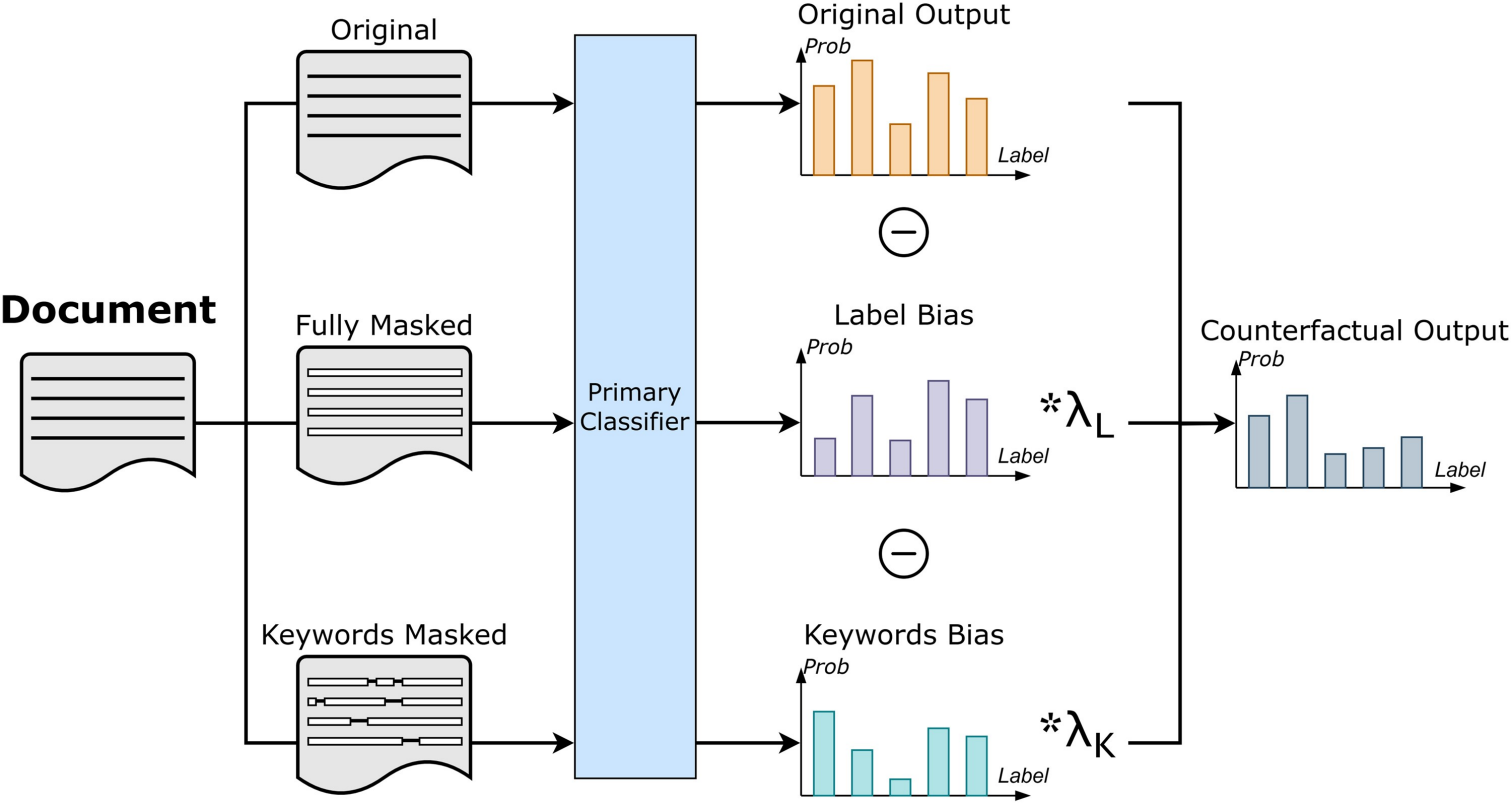
Overview of the post-processing step.

For medical-related texts, interpretability is of paramount importance. Therefore, we employ a counterfactual postprocessing method to further address bias (Qian *et al.* 2021). In this approach, both label bias and keyword bias are considered. As shown in Fig. 2, to compute label bias, the entire document content is masked, and the resulting probability distribution from the model’s output serves as the label bias. For keyword bias, all content except for the keywords is masked, and the resulting probability distribution represents the keyword bias. The output after applying the postprocessing step is computed as follows:


(8) $$O(x)=f_{M_{1}}(x)-\lambda_{L}f_{M_{1}}(x_{fm})-\lambda_{K}f_{M_{1}}(x_{km}),$$

where $x_{fm}$ is the fully masked input, $x_{km}$ is the keyword-masked input, and $\lambda_{L}$, $\lambda_{K}$ are additional parameters, which are determined by searching for the values that maximize the performance metrics on the validation set.

This postprocessing step improves interpretability by making the source of bias explicitly measurable. By comparing model outputs on (i) the original input, (ii) a fully masked input, and (iii) a keyword-only input, we can directly quantify how much of a prediction is attributable to label priors or specific keywords rather than contextual semantics. The final adjustment in Equation (8) subtracts these quantified bias components, allowing the model to correct predictions while also making the contribution of bias transparent and explainable at the instance level.

**Algorithm 1**  **Framework of CoDeNet** **Require:** training set $D_{T}$, validation set $D_{V}$, learning rate η, number of warm up iterations $T_{w}$, number of iterations *T*, loss weight function W, fully mask function FM, keywords mask function KM, metric function M;

1: Initialize the primary classifier $f_{M_{1}}(x;\theta_{M_{1}})$.;

2: **for**  $t=\begin{array}{c} 1,\ldots ,T_{w} \end{array}$  **do** 

3:  Draw a mini-batch $B={\left\{(x^{\left(b\right)},y^{\left(b\right)})\right\}}_{b=1}^{B}$ from $D_{T}$.;

4:  $\theta_{M_{1}}\leftarrow \theta_{M_{1}}-\eta \nabla_{\theta_{M_{1}}}\sum_{(x,y)\in B}\text{CE}(f_{M_{1}}(x),y)$;

5: **end for** 

6: Initialize the debias estimator $f_{M_{2}}(x;\theta_{M_{2}})$.;

7: **for**  $t=\begin{array}{c} 1,\ldots ,T \end{array}$  **do** 

8:  Draw a mini-batch $B={\left\{(x^{\left(b\right)},y^{\left(b\right)})\right\}}_{b=1}^{B}$ from $D_{T}$.;

9:  $\theta_{M_{1}}\leftarrow \theta_{M_{1}}-\eta \nabla_{\theta_{M_{1}}}\sum_{(x,y)\in B}W(x)\text{CE}(f_{M_{1}}(x),y)$;

10:  $\theta_{M_{2}}\leftarrow \theta_{M_{2}}-\eta \nabla_{\theta_{M_{2}}}\sum_{(x,y)\in B}\text{GCE}(f_{M_{2}}(x),y)$;

11: **end for** 

12: Initialize empty dataset $D_{V}^{\prime }$.;

13: **for**  $(x,y)\in D_{V}$  **do** 

14:  $x_{fm}\leftarrow FM(x)$.;

15:  $x_{km}\leftarrow KM(x)$.;

16:  Add $\left(x,x_{fm},x_{km},y\right)$ to $D_{V}^{\prime }$.;

17: **end for** 

18: $\lambda_{L},\lambda_{K}\leftarrow {\text{argmax}}_{\lambda_{L},\lambda_{K}}\sum_{D_{V}^{\prime }}M(O(x,\lambda_{L},\lambda_{K}),y)$;

### 2.5 Summary of the proposed method

The proposed CoDeNet framework is designed to enhance multilabel text classification through a cooperative training approach followed by a counterfactual postprocessing step. The framework consists of three main stages: warm-up training, cooperative training, and postprocessing parameter optimization.

In the initial stage, the primary model is trained using conventional supervised learning to establish a solid baseline performance. Specifically, for $T_{w}$ iterations, primary classifier is optimized using the standard CE loss on the training dataset.

Following warm-up training, a debias estimator is introduced, which shares the same architecture as the primary classifier but is trained using the GCE loss. This step aims to help the primary classifier focus more on learning from informative samples. The training involves updating both models iteratively over *T* iterations. The optimization for the primary classifier incorporates a dynamic weight function W(x) to emphasize effective samples.

To further mitigate bias interpretably, a counterfactual debiasing method is applied in the postprocessing stage. The bias is quantified based on two factors: label bias and keyword bias.

Overall, the CoDeNet framework effectively leverages cooperative training to enhance learning from informative samples, while counterfactual postprocessing mitigates biases interpretably, resulting in a more robust multilabel classification model.

## 3 Results

In this section, we present a comprehensive evaluation of our proposed method through a series of experiments conducted on two benchmark datasets: BDI-Sen and DepressionEMO. The experimental process is structured as follows: first, we outline the experimental setup, including the configuration of baselines and evaluation metrics. Subsequently, we perform an in-depth analysis of the results, with particular emphasis on examining the influence of two critical hyperparameters—the classifier’s hidden size and the *q*-value in GCE loss—on model performance, as these parameters are identified as significant determinants of our method’s effectiveness. To further understand the contribution of each component, we conduct an ablation study that systematically evaluates the impact of individual elements.

### 3.1 Datasets

The proposed method is validated utilizing two multilabel datasets, the DepressionEMO (Rahman *et al.* 2024) dataset and the BDI-Sen (Pérez *et al.* 2023) dataset. The documents of the DepressionEMO dataset are the user posts collected from different depression-related subreddits on the Reddit website, i.e. r\depression, r\partners, r\loneliness, r\suicide, and r\suicide_watch. The annotators systematically focused on eight emotions as labels, which are rigorously derived from established psychiatric criteria for depression, particularly those delineated by the American Psychiatric Association. (American Psychiatric Association: What is depression? Available at: https://www.psychiatry.org/patients-families/depression/what-is-depression.) The documents in the BDI-Sen dataset are selected from the eRisk2019 training set (Losada *et al.* 2019). They were annotated in accordance with the BDI-II questionnaire (Beck *et al.* 1996), which encompasses 21 recognized symptoms, spanning emotional, cognitive, and physical markers.

Both datasets exhibit substantial label imbalance, creating realistic conditions for studying the impact of sample-dependent bias. In DepressionEMO, the most frequent label (*sadness*) occurs approximately four times more often than the least common label [*brain dysfunction* (*forget*)]. The imbalance in BDI-Sen is even more pronounced, where the most frequent label (*Sadness*) appears roughly 65 times more often than the least frequent label (*Loss_of_interest_in_sex*). Such long-tailed distributions can lead models to disproportionately favor majority labels during training.

In addition, keyword bias caused by label-associated lexical patterns is also present in both datasets. A brief qualitative analysis is provided in the Supplementary Material, available as supplementary data at *Bioinformatics* online. To demonstrate that our method remains effective across a broader range of biomedical applications, we also additionally conduct experiments on the MIMIC-III dataset in the Supplementary Material, available as supplementary data at *Bioinformatics* online.

### 3.2 Baselines

To comprehensively evaluate the performance of our proposed method, we compare it with a series of baseline models, which can be broadly categorized into machine learning models and deep learning models. All baseline models follow the binary relevance framework, where a separate classifier is trained for each label independently.

For machine learning models, we use term frequency-inverse document frequency features to represent the input text. These features serve as input for classic machine learning algorithms, including logistic regression (LR) (Hosmer and Lemeshow), support vector machine (SVM) (Cortes and Vapnik 1995), XGBoost (Chen *et al.* 2016), and LightGBM (Ke *et al.* 2017).

As mentioned previously, we adopt pretrained transformer-based encoders to encode the input text for deep learning models. To conserve memory during training, all labels share a single encoder. Specifically, we use BERT (Devlin *et al.* 2019) as the primary transformer-based encoder. To assess the scalability and robustness of our method, we experiment with three BERT variants of different sizes: BERT-mini, BERT-base, and BERT-large. Furthermore, we explore the impact of domain-specific knowledge captured in the pretraining step. For this purpose, we include MentalBERT (Ji *et al.* 2022).

Below are the details of the baseline models used in our experiments:

- LR: Logistic regression applies a logistic function to the output of a linear equation to produce a probability between 0 and 1.
- SVM: SVM works by finding a hyperplane that best separates the data points of different classes in a high-dimensional feature space.
- XGBoost: XGBoost builds an ensemble of decision trees sequentially, where each new tree attempts to correct the errors (residuals) of the previous trees by focusing on difficult-to-predict samples.
- LightGBM: LightGBM builds an ensemble of decision trees in a leafwise fashion, which means it splits the tree nodes with the maximum gain at each step, as opposed to the levelwise approach used in traditional gradient boosting methods. This often leads to deeper, more accurate trees with fewer iterations. LightGBM also employs histogram-based techniques to speed up the training process, reducing memory consumption and improving training time.
- BERT: BERT builds a pretrained bidirectional text encoder that helps the classifier capture richer contextual information.
- MentalBERT: MentalBERT is pretrained on clinical notes, psychiatric evaluations, mental health forums, and other relevant resources, allowing it to learn the unique language and terminology used in mental health contexts.

In addition to varying the base models, we also compare our method with traditional debiasing approaches in the Supplementary Material, available as supplementary data at *Bioinformatics* online.

### 3.3 Evaluation metrics

In this study, we evaluate the performance of multilabel text classification using two commonly adopted metrics: F1-macro and F1-micro. These metrics are selected because they provide complementary insights into model performance for multilabel classification tasks.

F1-micro aggregates the contributions of all labels to compute a single F1 score by considering the total number of true positives, false positives, and false negatives across all labels. This metric is particularly suitable when labels are imbalanced, as it focuses on the overall prediction performance, giving more weight to the most frequent labels. Specifically, the formulas are:


(9) $$\text{Precision}=\frac{\sum_{i=1}^{L}TP_{i}}{\sum_{i=1}^{L}\left(TP_{i}+FP_{i}\right)},$$

(10) $$\text{Recall}=\frac{\sum_{i=1}^{L}TP_{i}}{\sum_{i=1}^{L}\left(TP_{i}+FN_{i}\right)},$$

(11) $$F1-\text{micro}=\frac{2\times \text{Precision}\times \text{Recall}}{\;\text{Precision}+\text{Recall}}.$$

F1-macro, on the other hand, calculates the F1 score for each label independently and then takes the average. Unlike F1-micro, it treats all labels equally, regardless of their frequency. This makes F1-macro more sensitive to the model’s performance on minority labels, providing a balanced view of how well the model generalizes across all labels. Specifically, the formulas are:


(12) $${F1-\text{score}}_{i}=2\times \frac{{\text{Recall}}_{i}\times {\text{Precision}}_{i}}{{\text{Recall}}_{i}+{\text{Precision}}_{i}},$$

(13) $$F1-\text{macro}=\frac{1}{L}\sum_{i=1}^{L}{F1-\text{score}}_{i}.$$

By combining F1-micro and F1-macro, we can obtain a comprehensive evaluation of the model: F1-micro highlights the overall prediction accuracy, while F1-macro ensures that performance on less frequent labels is not overlooked. These metrics allow us to assess both the robustness and fairness of the proposed method across different datasets and label distributions.

To provide statistically reliable comparisons and meet reproducibility expectations, we additionally report the 95% confidence intervals of model performance for both the deep learning baselines and their versions enhanced with our method. Confidence intervals are computed using nonparametric bootstrap resampling. The mean and 95% confidence bounds are shown in Table 1. This protocol allows us to assess whether the observed performance gains are statistically meaningful rather than arising from dataset-specific randomness.

**Table 1:** Base experiment.

Model	DepressionEMO		BDI-Sen	
	F1_micro (%)	F1_macro (%)	F1_micro (%)	F1_macro (%)
BERT-mini	75.67±1.22	65.73±1.18	17.81±2.91	19.34±3.25
BERT-base	80.03±1.16	75.63±1.40	48.36±5.34	54.37±5.98
BERT-large	80.86±1.18	75.34±1.57	46.99±5.79	53.26±6.83
MentalBERT	80.25±1.17	73.62±1.45	53.51±5.50	59.97±6.45
LR	74.82	64.39	29.42	9.66
SVM	74.29	65.08	51.39	28.27
XGBoost	73.36	64.93	53.47	47.61
Light GBM	72.99	64.24	49.55	44.37
BERT-mini w/ours	76.20±1.23	66.50±1.34	48.42±5.34	47.46±5.63
BERT-base w/ours	81.51±1.13	77.79±1.40	60.21±5.30	60.94±6.39
BERT-large w/ours	81.12±1.14	76.57±1.47	58.63±5.70	58.31±6.34
MentalBERT w/ours	80.50±1.17	74.63±1.52	56.92±5.51	57.44±5.86

### 3.4 Base experiment results

Our proposed method is applied to deep learning models, and the experimental results are summarized in Table 1.

For the DepressionEMO dataset, BERT-based models outperform traditional machine learning models such as LR, SVMs, XGBoost, and LightGBM by a significant margin. Among the BERT models, BERT-large achieves the highest F1-micro score of 80.86%, demonstrating strong predictive capability. BERT-base and MentalBERT also perform well, with MentalBERT showing a more balanced performance across both metrics due to its mental health-specific pretraining. In the BDI-Sen dataset, traditional machine learning models struggle, especially in terms of F1-macro, reflecting their limited ability to capture minority labels in complex multilabel tasks. Among the deep learning baselines, MentalBERT again delivers the best results, with an F1-micro score of 53.51% and an F1-macro score of 59.97%. This highlights the advantage of domain-specific pretraining for improving generalization on specialized datasets.

It is important to note that, unlike the baselines provided by DepressionEMO (Rahman *et al.* 2024), which transform multilabel classification into a multiclass problem by treating each label combination as a distinct class, we classify each label independently using the BR approach. This method effectively reduces the class imbalance issue encountered in multiclass transformations, resulting in better performance for the same baseline models in our experiments.

Our proposed method significantly improves the performance of all deep learning models across both datasets. In the DepressionEMO dataset, BERT-base with our method achieves the best results, with an F1-micro of 81.51% and an F1-macro of 77.79%, representing a substantial improvement over the baseline. The smallest model, BERT-mini model, also benefits from our approach, improving its F1-macro score from 65.73% to 66.50%. The BDI-Sen dataset shows even more significant improvements. The F1-micro score of BERT-base increases from 48.36% to 60.21%, while its F1-macro rises from 54.37% to 60.94%. BERT-large and MentalBERT also show notable improvements, with MentalBERT achieving an F1-micro of 56.92% and an F1-macro of 57.44%. These improvements demonstrate that our method effectively addresses the bias caused by the imbalance of the dataset and enhances the overall performance of the model.

### 3.5 Impact of hidden size of the classifier

The hidden size refers to the number of units in the hidden layer of the classifier. When the hidden size is set to 0, the classifier becomes a linear layer without any hidden layer units. This experiment evaluates whether increasing classifier complexity provides meaningful benefits beyond the semantic representations already produced by the transformer encoder.

As shown in Table 2, the performance across both datasets varies considerably with different hidden sizes. For the DepressionEMO dataset, the best performance is observed when the hidden size is set to 64, with F1-micro and F1-macro scores of 81.45% and 77.76%, respectively. Interestingly, the hidden size of 0 also achieves competitive results (81.30% F1-micro and 77.10% F1-macro). However, as the hidden size increases beyond 128, the performance starts to decline. For the BDI-Sen dataset, the hidden size of 0 achieves the best results, with an F1-micro of 60.50% and F1-macro of 62.41%. As the hidden size increases, the performance drops sharply. For instance, the F1-micro score decreases from 51.12% at size 64 to just 27.64% at size 448. The model performance on both datasets indicates that a simpler classifier can generalize well for them.

**Table 2:** Experiment for impact of hidden size.

Hidden size	DepressionEMO		BDI-Sen	
	F1_micro (%)	F1_macro (%)	F1_micro (%)	F1_macro (%)
0	81.30	77.10	60.50	62.41
64	81.45	77.76	51.12	54.41
128	80.29	73.28	45.07	51.14
192	81.03	77.45	42.66	50.23
256	79.86	72.55	35.64	46.60
320	80.31	72.06	37.96	42.39
384	80.88	73.06	34.20	45.41
448	80.46	71.67	27.64	40.45
512	79.90	72.66	31.35	45.25

### 3.6 Impact of *q*-value in GCE loss

The hyperparameter *q*-value in the GCE loss plays a critical role in controlling the focus of the model during training. Specifically, higher values of *q* emphasize the learning of easier-to-classify samples. As shown in Table 3, increasing *q*-value leads to a significant decline in performance, particularly for datasets with imbalanced label distributions, such as BDI-Sen.

**Table 3:** Experiment for impact of *q*-value in GCE loss.

q	DepressionEMO		BDI-Sen	
	F1_micro (%)	F1_macro (%)	F1_micro (%)	F1_macro (%)
0.1	81.45	77.76	60.50	62.41
0.2	81.11	76.97	57.23	56.62
0.3	80.22	75.69	55.94	56.60
0.4	79.84	71.39	55.76	56.97
0.5	78.07	65.67	59.56	59.37
0.6	77.50	66.49	57.44	58.35
0.7	77.56	66.19	58.92	60.56
0.8	75.86	65.30	56.11	56.34
0.9	75.21	64.57	58.21	60.50
1.0	74.99	64.18	55.56	58.46

These results provide strong evidence for our previous hypothesis: GCE loss serves as an excessive correction mechanism, which can be detrimental to model performance when the correction is too aggressive. A high *q*-value increases the tendency of the debias estimator to ignore less representative samples. While this is designed to reduce the impact of noisy or ambiguous labels, it also discards potentially valuable information contained in those less common samples. To further validate this, we conducted additional experiments, as presented in Table 4, demonstrating that taking the debias estimator as the classifier directly would receive worse performance.

**Table 4:** Classification performance of debias estimator.

Backbone	DepressionEMO		BDI-Sen	
	F1_micro (%)	F1_macro (%)	F1_micro (%)	F1_macro (%)
BERT-mini	75.88	66.39	34.80	40.17
BERT-base	80.61	76.04	48.92	52.22
BERT-large	80.94	76.52	27.14	32.76
MentalBERT	81.03	76.95	57.96	61.64

Aside from MentalBERT, the performance of the other three models shows varying degrees of decline when applying GCE loss, particularly on the BDI-Sen dataset, where the drop is most pronounced. This discrepancy is likely due to the higher number of labels in BDI-Sen, which makes the dataset more complex and prone to information loss when GCE loss overly penalizes less representative samples.

### 3.7 Ablation experiment

The ablation experiment is conducted to evaluate the contributions of different components of our proposed method. We systematically analyze the impact of the cooperative training step and the postprocessing step by evaluating various configurations and comparing them to the baseline model.

#### 3.7.1 Impact of cooperative training

The first ablation scenario involves adding the cooperative training step to the baseline BERT-based model. As shown in Table 5, incorporating the cooperative training step leads to noticeable improvements across both the F1-micro and F1-macro metrics. Specifically, the F1-micro score increases from 80.05% to 81.12% on DepressionEMO and from 48.49% to 55.53% on BDI-Sen. Similarly, the F1-macro score improves from 75.68% to 77.12% on DepressionEMO and from 55.39% to 60.09% on BDI-Sen. These results demonstrate that the cooperative training step effectively aids in boosting the model’s performance, particularly in learning from more informative samples.

**Table 5:** Ablation experiment.

Model	DepressionEMO		BDI-Sen	
	F1_micro (%)	F1_macro (%)	F1_micro (%)	F1_macro (%)
Vanilla BERT	80.05	75.68	48.49	55.39
+ Cooperative training	81.12	77.12	55.53	60.09
+ Postprocessing	80.20	75.90	56.98	59.68
Cooperative training	81.45	77.76	60.50	62.41
+ Postprocessing				

#### 3.7.2 Impact of postprocessing

The second ablation configuration evaluates the influence of postprocessing. The results show a marginal improvement when postprocessing is added, with the F1-micro score on DepressionEMO increasing from 80.05% to 80.20%, and the F1-macro score rising from 75.68% to 75.90%. On BDI-Sen, F1-micro increases from 48.49% to 56.98%, and F1-macro increases from 55.39% to 59.68%. While the gains are less pronounced compared to the cooperative training step, the postprocessing step does contribute to refining the model’s predictions in an interpretable way.

#### 3.7.3 Combined effect of cooperative training and postprocessing

Finally, the combination of the cooperative training step and postprocessing results in the best performance across both datasets. As shown in the last row of Table 5, this configuration achieves an F1-micro score of 81.45% on DepressionEMO and 60.50% on BDI-Sen, and an F1-macro score of 77.76% on DepressionEMO and 62.41% on BDI-Sen. The results confirm that the synergistic effect of both components contributes significantly to improving the overall performance.

## 4 Discussion, and Conclusion

### 4.1 Discussion of experiment results

According to the results of the base experiment, the benefits of our method to model performance even surpass the influence of model scale and domain-specific knowledge, as the BERT-base model outperformed the BERT-large model and MentalBERT model on both datasets after applying our method. This suggests that our method assists the model in acquiring patterns that originally demanded a larger model size or domain-specific knowledge to be learned.

From the experiment of hidden size of the classifier, we observed that a simpler classifier can generalize well. A possible explanation for this phenomenon lies in the semantic richness of the encoder’s output representations. Pretrained transformer encoders such as BERT and MentalBERT provide highly contextualized and semantically rich representations of the input text, which already capture most of the essential information needed for classification. Therefore, adding a large hidden layer in the classifier may introduce unnecessary complexity, leading to a risk of overfitting or redundancy in the learned features.

The experiment of taking the debias estimator as the classifier directly presented an interesting phenomenon. MentalBERT appears to benefit from its domain-specific pretraining, which helps mitigate the loss of potentially useful but infrequent information. The incorporation of prior knowledge from the mental health domain may enable MentalBERT to compensate for the negative effects of excessive correction, leading to a more effective fine-tuning process when GCE loss is applied. However, despite this advantage, MentalBERT’s performance with GCE loss still does not surpass the best results obtained using our proposed method. This finding highlights a key distinction:

- MentalBERT’s domain knowledge is broad, encompassing general mental health information learned during pretraining.
- Our method enables models to acquire task-specific knowledge, ensuring that the learned representations are more aligned with the characteristics of the target dataset.

This phenomenon reinforces the effectiveness of our approach in guiding the model toward learning information truly relevant to the current task, rather than relying solely on pretrained domain knowledge, which may not always be optimal for the specific dataset at hand.

### 4.2 Innovation

CoDeNet introduces three key innovations. First, the cooperative training framework synergizes a primary classifier and a debias estimator, enabling dynamic reweighting of training samples based on their bias contributions. This approach contrasts with static reweighting methods, which often fail to adapt to evolving bias patterns during training. Second, the elastic scaling mechanism prevents overcorrection by leveraging the debias estimator as an auxiliary model rather than directly using it as the final classifier. This ensures that the primary classifier benefits from bias correction without being overly influenced by aggressive corrections, thereby preserving clinically meaningful signals. Third, the counterfactual post-processing step offers a novel, interpretable debiasing strategy by quantifying label and keyword biases through input masking. Unlike adversarial methods that risk suppressing critical features, this approach transparently isolates and mitigates biases without altering the core model architecture. These innovations collectively enable CoDeNet to outperform domain-specific models like MentalBERT, even without specialized pretraining, as evidenced by its superior F1-macro scores (77.79% versus 73.62% on DepressionEMO).

### 4.3 Limitations and future work

While CoDeNet exhibits strong performance, there are several areas for improvement. First, the counterfactual postprocessing step introduces additional computational overhead during inference. The inference time of the model that applied our method was approximately three times that of the original one, which may hinder real-time deployment in clinical settings. Future work will explore lightweight approximations or parallel processing techniques to mitigate this issue. Second, the current framework assumes that biases can be effectively identified through keyword masking, which may not capture all spurious correlations in complex clinical narratives. Integrating additional contextual information or leveraging external knowledge bases could further enhance bias detection and mitigation. Finally, extending CoDeNet to federated learning scenarios, where data privacy and security are paramount, represents a promising direction for future research. This would enable the framework to be applied across multiple institutions while maintaining data confidentiality.

### 4.4 Conclusion

Our proposed framework presents a robust and practical solution for multilabel text classification challenges, particularly in scenarios with imbalanced label distributions. The proposed framework not only advances current methodological approaches but also opens new avenues for future research, including potential applications in diverse domains and the integration of advanced debiasing techniques to further enhance model performance and fairness.

## Supplementary Material

btag317_Supplementary_Data

## Data Availability

The source code and implementation details of CoDeNet, including experimental data, will be publicly available on GitHub: https://github.com/66ccff39C5BB/CoDeNet.
