# Supplementary material for: Mitigating bias in multilabel medical text classification: a cooperative training framework with dynamic debiasing
Source: Bioinformatics. 2026 May 18;42(6):btag317. doi: 10.1093/bioinformatics/btag317 (PMC13292148; doi:10.1093/bioinformatics/btag317)
Supplement: btag317_Supplementary_Data [file btag317_supplementary_data.pdf]

Table 1. Results on MIMIC-III

Model	F1_micro(%)	F1_macro(%)
BERT-base	$40.79_{\pm 2.67}$	$32.12_{\pm 2.68}$
BioBERT	$38.70_{\pm 2.72}$	$30.89_{\pm 2.64}$
ClinicalBERT	$36.71_{\pm 2.58}$	$27.10_{\pm 2.26}$
RoBERTa-base	$38.92_{\pm 2.55}$	$28.76_{\pm 2.20}$
BERT-base w/ ours	$40.15_{\pm 2.13}$	$36.85_{\pm 2.21}$
BioBERT w/ ours	$41.06_{\pm 2.53}$	$31.82_{\pm 2.62}$
ClinicalBERT w/ ours	$40.04_{\pm 2.40}$	$27.53_{\pm 2.10}$
RoBERTa-base w/ ours	$41.09_{\pm 2.27}$	$31.54_{\pm 2.21}$

Table 2. Comparison of Different Debiasing Methods

Dataset	Method	F1_micro(%)	F1_macro(%)
DepressionEMO	IPS	$79.37_{\pm 1.17}$	$75.76_{\pm 1.39}$
	CORSAIR	$80.32_{\pm 1.16}$	$76.02_{\pm 1.44}$
	CoDeNet	$81.51_{\pm 1.13}$	$77.79_{\pm 1.40}$
BDI-Sen	IPS	$6.81_{\pm 1.25}$	$29.22_{\pm 5.22}$
	CORSAIR	$58.13_{\pm 5.39}$	$58.99_{\pm 5.94}$
	CoDeNet	$60.21_{\pm 5.30}$	$60.94_{\pm 6.39}$
MIMIC-III	IPS	$40.36_{\pm 2.47}$	$33.79_{\pm 2.65}$
	CORSAIR	$41.08_{\pm 2.70}$	$30.34_{\pm 2.54}$
	CoDeNet	$40.15_{\pm 2.13}$	$36.85_{\pm 2.21}$

Related Work

Multi-label Text Classification

Multi-label text classification (MLTC) extends traditional text classification by allowing each document to be associated with multiple labels, in contrast to single-label classification, where each document belongs to only one category. This problem has garnered significant attention in recent years due to the increasing complexity of real-world textual data, as observed in applications such as news categorization, medical diagnosis, and sentiment analysis, where a single label is often insufficient to fully capture the semantic content of a document.

Neural network-based models have demonstrated superior performance in MLTC tasks. Recurrent neural networks (RNNs) and convolutional neural networks (CNNs) have been widely adopted for processing textual data(Zhang and Zhou, 2013). Chen et al. proposed an LSTM-based approach to capture contextual dependencies between textual features and labels, enhancing classification effectiveness (Chen et al., 2018).

MLTC has undergone substantial advancements, driven by methodological innovations and the adoption of deep learning architectures. Despite persistent challenges related to label imbalance, inter-label dependencies, and model interpretability, ongoing research continues to address these issues through novel algorithmic strategies and domain-specific adaptations.

Debiasing Methods

Bias in machine learning models has garnered significant attention due to its profound implications for fairness and reliability. A variety of approaches have been proposed to mitigate bias, which can be broadly classified into pre-processing, in-processing, and post-processing strategies(Barocas et al., 2023; Mehrabi et al., 2021). These techniques seek to address biases in the data, the algorithms, and the resulting predictions, respectively.

In-processing methods modify the model training process itself to alleviate bias. A prominent approach involves the incorporation of fairness constraints within the objective function. Hardt et al. (Hardt et al., 2016) introduced the equalized odds framework, which adjusts decision thresholds to balance false positive and false negative rates across groups. Zafar et al. (Zafar et al., 2017) proposed fairness-aware optimization by imposing constraints on loss functions to enforce fairness conditions. Another notable in-processing technique is adversarial training, wherein Zhang et al. (Zhang et al., 2018) developed an adversarial model that promotes fairness by minimizing the ability of an auxiliary network to predict sensitive attributes.

Post-processing methods aim to adjust the model's outputs after training to reduce bias. One of the earliest such methods, introduced by Hardt et al. (Hardt et al., 2016), modifies decision thresholds to ensure fairness across groups. Other post-processing strategies include confidence calibration (Wei et al., 2020) and re-ranking algorithms (Liu and Burke, 2018) designed to guarantee equitable treatment among demographic groups.

Despite these advancements, challenges persist in balancing fairness with model accuracy, ensuring generalizability across diverse domains, and addressing intersectional biases.

Evaluation on the MIMIC-III Dataset

Our original dataset selection was focused on depression detection. To further demonstrate the robustness and generalizability of CoDeNet on a widely used medical benchmark, we supplemented experiments on the MIMIC-III dataset according to reviewer suggestions. MIMIC-III is a large-scale clinical database commonly used for automated ICD coding tasks, thus providing a strong testbed for validating the effectiveness of debiasing methods in real-world hospital records.

Due to time and computational constraints, we sampled a subset of the corpus and randomly selected ten ICD-9 codes as target labels. We followed the same training and evaluation protocols as in the main experiments and adopted several widely used biomedical pre-trained models, including BERT-base, BioBERT, ClinicalBERT, and RoBERTa-base. Their corresponding CoDeNet-enhanced variants were also evaluated, and all results are reported with 95% confidence intervals computed via non-parametric bootstrap. The results are shown in Table 1.

Across most settings, the application of our method improves performance, particularly in macro-F1, suggesting better handling of low-frequency diagnostic codes. This is consistent with observations from the DepressionEMO and BDI-Sen datasets, indicating that CoDeNet can mitigate biases induced by label imbalance even on more conventional medical datasets.

Comparison with Other Debiasing Methods

To further evaluate the effectiveness of CoDeNet and situate it relative to representative debiasing strategies, we additionally compare our method with two widely examined approaches in existing literature:

- **Inverse Propensity Scoring (IPS)**, a reweighting technique commonly adopted in recommender systems to correct exposure bias by adjusting sample loss weights based on inverse label frequency(Schnabel et al., 2016). We adapt IPS to the multi-label classification setting by computing class-dependent inverse propensity scores and applying them as dynamic weights in the loss function.

- **CORSAIR**, a counterfactual reasoning framework that corrects prediction bias by perturbing key feature components, and represents the class of counterfactual post-hoc debiasing strategies(Qian et al., 2021).

These two baselines correspond to the major lines of research discussed in the main paper: IPS represents static statistical reweighting methods, and CORSAIR represents counterfactual approaches that adjust predictions after inference. In contrast, CoDeNet integrates debiasing into the training dynamics by allowing a debias estimator to adaptively emphasize samples that are predicted too confidently, mitigating the risk of shortcut learning without relying exclusively on external priors or synthetic counterfactual augmentation.

We evaluate all methods on the DepressionEMO, BDI-Sen, and MIMIC-III datasets under identical training protocols. The experimental results with 95% confidence intervals are reported in Table 2. The comparative results in Table 2 reveal distinct performance patterns across the three datasets. CoDeNet achieves superior or highly competitive performance, particularly on the macro-F1 score, which is crucial for imbalanced medical datasets.

On the DepressionEMO and MIMIC-III datasets, all three methods demonstrate comparable performance in micro-F1, suggesting a baseline level of effectiveness on the majority classes. However, the advantage of CoDeNet becomes evident in the macro-F1 metric. On DepressionEMO, CoDeNet outperforms both baselines, indicating a more balanced performance across all classes. This trend is more pronounced on MIMIC-III, where CoDeNet achieves a significantly higher macro-F1 (36.85%) compared to IPS (33.79%) and CORSAIR (30.34%). This demonstrates that CoDeNet's cooperative training framework successfully mitigates spurious correlations without disproportionately harming the classification of tail classes, a common pitfall of some debiasing methods.

The results on the BDI-Sen dataset require a more nuanced discussion. While CORSAIR and CoDeNet show substantial and comparable improvements over a naive baseline, the IPS method fails dramatically, with a micro-F1 of only 6.81%. This anomaly is instructive and can be attributed to a fundamental limitation of IPS. The effectiveness of IPS is highly sensitive to the accuracy of the propensity scores. In a noisy, high-dimensional text domain with extreme class imbalance, these scores are difficult to estimate reliably. An inaccurate propensity model can assign excessively large weights to a small subset of noisy or hard samples, destabilizing the training process and causing the model to converge to a degenerate solution. This aligns with our earlier motivation—IPS, as a static reweighting method, lacks the adaptive, data-driven reasoning about how bias manifests in individual examples during training.

In contrast, CoDeNet is designed to circumvent this limitation. Instead of relying on a fixed, pre-computed prior like IPS, the debias estimator in CoDeNet learns to quantify bias dynamically in cooperation with the primary classifier. This allows the framework to adaptively adjust the learning signals without being derailed by inaccurate propensity estimates. The strong and stable performance of CoDeNet on BDI-Sen, alongside CORSAIR, validates the rationale behind integrating adaptive, data-driven debiasing directly into the training loop. It confirms that our method does not merely capitalize on the failure of a weak baseline but offers a robust and principled alternative that addresses the core limitations of previous paradigms.

Qualitative Analysis

Our post-processing method explicitly eliminates keyword bias; however, through experiments, we observed that the cooperative training process also implicitly alleviates the keyword bias problem in the model. To demonstrate this, we analyze the attention weights in the attention layers of the BERT encoder. Specifically, we focus on the attention weights of the '[CLS]' token for other tokens, averaged across all layers and heads, and then scaled by a factor of 100 for visualization purposes.

Fig. 1 presents attention weights for six examples (three from each dataset) comparing the Vanilla BERT encoder and the BERT encoder trained with our proposed method. The comparison reveals that our method increases the attention weights assigned to label-related keywords while decreasing the weights for label-unrelated keywords.

This phenomenon can be attributed to the cooperative training mechanism, which enhances the model's ability to focus on informative content. By emphasizing learning from effective information during training, the model is better equipped to identify text patterns relevant to the target labels, distinguishing meaningful keywords from distracting ones. Such improvements in attention distribution demonstrate the efficacy of our approach in addressing keyword bias both explicitly and implicitly.

References

S. Barocas, M. Hardt, and A. Narayanan. *Fairness and machine learning: Limitations and opportunities*. 2023.

Y. Chen, B. Xiao, Z. Lin, C. Dai, Z. Li, and L. Yan. Multi-label text classification with deep neural networks. In *2018 International Conference on Network Infrastructure and Digital Content (IC-NIDC)*, pages 409–413. IEEE, 2018.

M. Hardt, E. Price, and N. Srebro. Equality of opportunity in supervised learning. *Advances in neural information processing systems*, 29, 2016.

W. Liu and R. Burke. Personalizing fairness-aware re-ranking. *arXiv preprint arXiv:1809.02921*, 2018.

N. Mehrabi, F. Morstatter, N. Saxena, K. Lerman, and A. Galstyan. A survey on bias and fairness in machine learning. *ACM computing surveys (CSUR)*, 54(6):1–35, 2021.

C. Qian, F. Feng, L. Wen, C. Ma, and P. Xie. Counterfactual inference for text classification debiasing. In *Proceedings of the 59th Annual Meeting of the Association for Computational Linguistics and the 11th International Joint Conference on Natural Language Processing (Volume 1: Long Papers)*, pages 5434–5445, 2021.

T. Schnabel, A. Swaminathan, A. Singh, N. Chandak, and T. Joachims. Recommendations as treatments: Debiasing learning and evaluation. In *international conference on machine learning*, pages 1670–1679. PMLR, 2016.

D. Wei, K. N. Ramamurthy, and F. P. Calmon. Optimized score transformation for fair classification. *Proceedings of Machine Learning Research*, 108, 2020.

M. B. Zafar, I. Valera, M. G. Rogriguez, and K. P. Gummadi. Fairness constraints: Mechanisms for fair classification. In *Artificial intelligence and statistics*, pages 962–970. PMLR, 2017.

B. H. Zhang, B. Lemoine, and M. Mitchell. Mitigating unwanted biases with adversarial learning. In *Proceedings of the 2018 AAAI/ACM Conference on AI, Ethics, and*

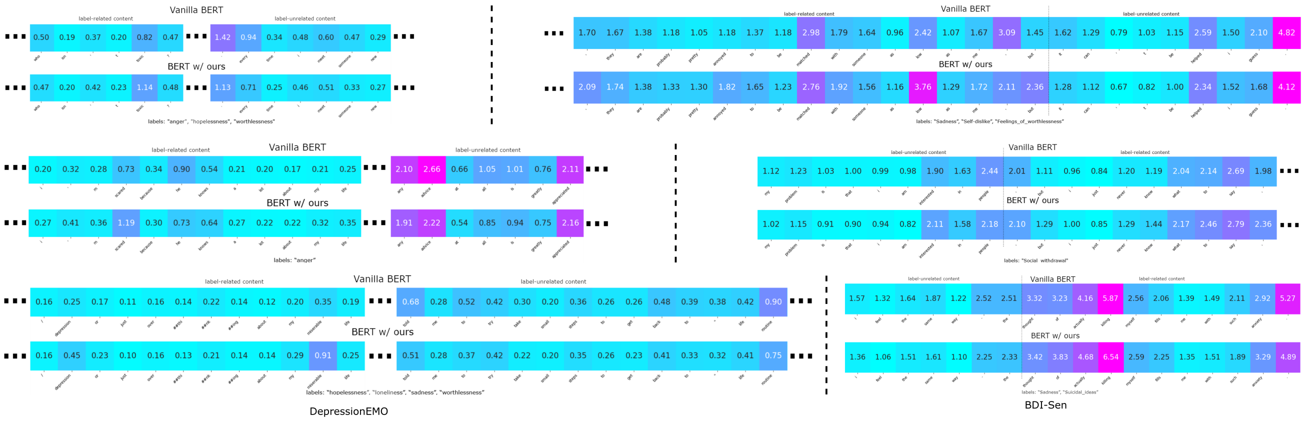

Fig. 1. Qualitative analysis for the effect of keyword debiasing

Society, pages 335–340, 2018.

M.-L. Zhang and Z.-H. Zhou. A review on multi-label learning algorithms. *IEEE transactions on knowledge and data engineering*, 26(8):1819–1837, 2013.
